# Volume-outcome relationship in rectal cancer surgery

**DOI:** 10.1007/s12672-021-00406-9

**Published:** 2021-04-12

**Authors:** L. Siragusa, B. Sensi, D. Vinci, M. Franceschilli, C. Pathirannehalage Don, G. Bagaglini, V. Bellato, M. Campanelli, G. S. Sica

**Affiliations:** Department of Surgical Science, University Tor Vergata, Viale Oxford 81, 00133 Rome, Italy

**Keywords:** Rectal cancer, Volume/outcome, Anastomotic leak

## Abstract

**Introduction:**

Hospital centralization effect is reported to lower complications and mortality for high risk and complex surgery operations, including colorectal surgery. However, no linear relation between volume and outcome has been demonstrated. Aim of the study was to evaluate the increased surgical volume effect on early outcomes of patient undergoing laparoscopic restorative anterior rectal resection (ARR).

**Methods:**

A retrospective analysis of all consecutive patients undergoing ARR with primary anastomosis between November 2016 and December 2020 after centralization of rectal cancer cases in an academic Centre. Short-term outcomes are compared to those of patients operated in the same unit during the previous 10 years before service centralization. The primary outcome was estimated anastomotic leak rate. Mean operative time, need of conversion, postoperative use of blood transfusion, radicality, in-hospital stay, number and type of complications, readmission and reoperation rate, mortality and 1-year and stoma persistence rates were evaluated as secondary outcomes.

**Results:**

86 patients were operated in the study period and outcomes compared to those of 101 patients operated during the previous ten years. Difference in volume of surgery was significant between the two periods (p 0.019) and the estimated leak rate was significantly lower in the higher volume unit (p 0.047). Mean operative time, need of conversion, postoperative use of blood transfusion and in-hospital stay (p < 0.05) were also significantly reduced in Group A.

**Conclusion:**

This study suggests that the shift toward higher volume in rectal cancer surgery is associated to decreased anastomotic leak rate. Potentiation of lower volume surgical units may yield optimal perioperative outcomes.

## Introduction

The first article describing a relationship between volume and outcome was reported by Luft et al. in 1979 [1]. Since then, an increasing evidence suggests that a high surgical volume is a critical factor in improving post-operative and long-term outcomes for challenging oncological procedures such as esophagectomy, gastrectomy, pancreatectomy and hepatectomy [2–8]. Therefore, specific policies across several countries have been implemented to provide a better care by centralizing the provision of this procedures in high-volume hospitals [9, 10].

The same is not validated for rectal cancer where conflicting existing evidences make the volume/outcome relationship still debatable [11, 12].

Nevertheless, rectal cancer management is particularly challenging requiring a careful preoperative staging and multidisciplinary team discussion, essential to individualize the treatment to the many options available (neoadjuvant radio- chemotherapy, local excisions, surgery and watch and wait strategies) depending on patients, cancer stage and location [13]. In addition, the adoption of new techniques such as laparoscopic and robotic approach and fast-track protocols demonstrates to be safe and effective in guaranteeing equal oncological outcomes and some short-term advantages, with an eye to preserving better quality of life (QOL) [14–20].

The narrow pelvis anatomy makes rectal resection a complex surgery and noble structures proximity makes a challenge for surgeons to minimize morbidity while still achieving a good oncological outcome. Thus, morbidity and mortality in rectal cancer are still relatively high with anastomotic leakage remaining the most fearful complication characterized by long lasting clinical consequences, including mortality, significant impact on long-term functional and oncological outcomes and QOL [21–29].

While several authors have already compared low- and high-volume hospitals with conflicting results, the effect of increasing volume in a single institution has not been analyzed yet. Hence, the aim of this single-center study in which is evaluated the effect of surgical volume increase on anastomotic leakage and postoperative outcomes of patient undergoing restorative laparoscopic anterior rectal resection (ARR) for cancer.

## Materials and methods

### Study design and population

A single-centre retrospective study evaluating effect of service centralization on the perioperative outcomes of rectal cancer patients’ undergoing surgical resection.

In November 2016, colon and rectal cancer patients referred to our Institution were centralized in a newly designed Minimally Invasive Surgery Unit, under a single surgeon. The study involved all consecutive eligible patients undergoing elective restorative anterior rectal resection (ARR) for rectal cancer between November 2016 and December 2020 in the new unit (Group A).

Outcomes for Group A were compared with an historical control group, consisting of all consecutive patients undergoing ARR in the same hospital between January 2006 and October 2016 (Group B).

Data were extrapolated from a prospectively maintained database, recording continuous and discrete variables regarding biometric data, patient-related risk factors, preoperative blood test, tumor characteristics, neoadjuvant therapy, surgical approach, de-functioning stoma and outcomes.

During the postoperative period, any complication (intended as any adverse event during the follow-up period) including infective complications, anastomotic leak (“A defect of the intestinal wall at the anastomotic site leading to a communication between the intra- and extraluminal compartments) [30], surgical site infections (SSI, defined according to the Centre for Disease Control and Prevention, CDC/NHNS) [31], pneumonia (clinical symptoms, confirmed by radiological examination), ileus, bleeding, was recorded and graded according to Clavien-Dindo classification [32].

A scheduled Enhanced recovery after surgery (ERAS) protocol, based on the 2012 guidelines available at the time [33] was systematically applied during the study period. In the control group no ERAS protocol was formally in place although some items, such as avoidance of nasogastric tube and urinary catheter, early feeding, early mobilization and pre-operative thromboprophylaxis, were commonly applied.

### Inclusion and exclusion criteria

We included all patients aged above 18 years with a diagnosis of a cancer located in the rectum, defined according to the international definition by D’Souza et al. [34], scheduled for anterior rectal resection with primary anastomosis (with or without diverting loop ileostomy).

### Exclusion criteria

Exclusion criteria were inflammatory bowel disease, acquired or congenital immunodeficiency, preoperative infection, pregnancy, high anesthesiology risk (ASA IV), emergency surgery, presence of synchronous cancers, failure to perform rectal resection and primary anastomosis.

### Endpoints

This study’s primary outcome was to evaluate the estimated anastomotic leak rate difference amongst the two groups. Leaks were evaluated according to the grading of anastomotic leakage following anterior resection of the rectum proposed by the International Study Group of Rectal Cancer [35].

Secondary endpoints were: operative time, use of minimally invasive approaches, conversion to open surgery rate, postoperative use of blood transfusion, oncological radicality, postoperative length of stay (LOS), 30-days postoperative complications (as classified by Clavien-Dindo), surgical site infections, pneumonia, ileus, bleeding, reoperation, readmission, mortality and stoma persistence 1-year after surgery. All endpoints were analyzed in both group, A and B.

### Statistical analysis

Characteristics were summarized by means of the levels for categorical variables or by means of quantiles for continuous variables. Non-parametric tests were performed for comparisons between groups (Chi-Squared and Fisher Exact test in case of categorical variables, Wilcoxon test in case of continuous variables). Cox-Stuart test was used to test whether the data have an increasing or decreasing trend. Due to large temporal difference between groups A and B, a prediction of “surgical volume” and “leak” for the period 2021–2026 was made using a linear regression model bases on the data obtained in the period 2017–2020. All tests were 2-sided, accepting *p* < 0.05 as indicating a statistically significant difference and confidence intervals were calculated at 95% level. The analysis was performed using the R software (R Core Team (2020). R: A language and environment for statistical computing. R Foundation for Statistical Computing, Vienna, Austria. URL https://www.R-project.org/).

### Ethics

This study was conducted according to the international ethical recommendations on clinical research established by the Helsinki Declaration. The study was conducted in accordance with STROBE criteria (htpp://strobe-statement.org) and registered under clinicaltrials.gov: NCT04761536 [36].

## Results

### Study population

From January 2006 to December 2020, 203 consecutive patients diagnosed with rectal cancer scheduled for anterior rectal resection at Tor Vergata Hospital in Rome, Italy. Sixteen patients did not meet the inclusion criteria and were excluded from the analysis: five patients were affected by intestinal bowel diseases, one patient was classified at high risk for surgery (ASA 4), four patients had emergency surgery, six patients underwent a rectal resection without primary anastomosis.

One-hundred-eighty-seven patients undergoing elective ARR with primary anastomosis were subsequently included in the study analysis: eighty-six operated of ARR between November 2016 and December 2020 and treated following our ERAS protocol (Group A) and one-hundred-one operated of ARR between January 2006 and October 2016 and treated with conventional care. Patient selection is summarized in Table 1.

**Table 1:**
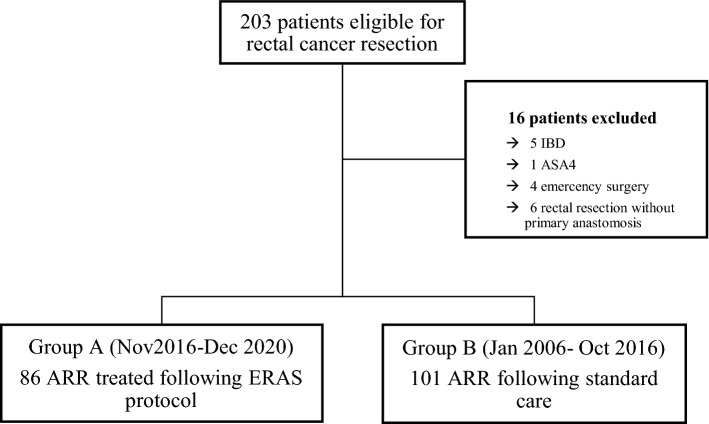
Patients selection

### Surgery volume

**Fig. 1:**
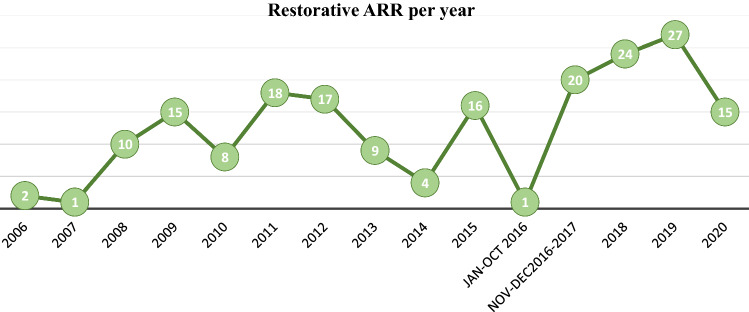
Volume of surgery per year

The number of procedures performed per year was significantly higher in the period November 2016-December 2020 (Group A) when compared to the period January 2006-October 2016 (Group B) with a mean procedure per year of 22 versus 9 respectively (p 0.019) (Table 2; Fig. 1).

**Table 2:** Volume of surgery

Hospital Volume	2006–2016, N = 11	2017–2020, N = 4	p-value 0.019	q-value^*2*^0.093
Procedure per year Mean	9 (7)	22 (5)		
Minimum, Maximum	1, 18	15, 27

### Baseline patient’s characteristic

The two groups were comparable with respect to age, sex, BMI, comorbidities, ASA score, preoperative albumin, distance from anal verge, neoadjuvant radiotherapy, mesorectal excision, defunctioning temporary stoma, positive lymph nodes (N) and distant metastasis (M). Preoperative hemoglobin was found to be higher in Group B with a mean of 13.38 ± 2.18 vs 12.42 ± 2.07 of Group A (p 0.003). Primary tumor extent (T) was significantly higher in Group A versus Group B (p 0.002). Baselines patients’ characteristics are summarized in Table 3.

**Table 3:** Baseline characteristics

Parameters	Group A (rectal cancer 2017–2020) (n = 86)	Group B (rectal cancer 2006–2016) (n = 101)	P
Age (mean, SD)	67.3 ± 12.2	67.94 ± 10.8	0.704
Sex %			0.460
Male	46–53.5%	60–59.4%	
Female	40–46.5%	41–39.6%	
Preoperative BMI (mean, SD)	25.6 ± 4.6	26,3 ± 4.2	0.278
ASA score %			0.407
1	14–16.2%	20–19.8%	
2	38–44.1%	49–48.5%	
3	34–39.5%	30–29.7%	
Morbidity %			
Diabetes	6–6.9%	14–13.8%	0.158
Hypertension	38–44.1%	51–50.5%	0.463
Cardiovascular disease	22–25.6%	21–20.8%	0.488
Respiratory disease	13–15.1%	10–9.9%	0.3721
Preoperative albumin (gr/dl) (mean, SD)	3.97 ± 0.51	3.87 ± 0.54	0.197
Preoperative hemoglobin (gr/dl) (mean, SD)	12.42 ± 2.07	13.38 ± 2.18	0.003
Distance from anal verge (cm) (mean, SD)	7.1 ± 2.8	6.7 ± 2.5	0.304
T %			0.002
1	10–11.6%	21–20.8%	
2	16–18.6%	17–16.8%	
3	39–45.4%	58–57.4%	
4a	14–16.3%	4–3.9%	
4b	7–8.1%	1–1%	
N + %	37–43%	36–35.6%	0.367
M + %	13–15.1%	7–6.9%	0.096
Neoadjuvant RT %	21–24.4%	27–26.7%	0.740
Surgical approach %			
Laparoscopy %	45–52.3%	20–19.8%	
Open	37–43%	73–72.3%	0.0002
Converted	4–4.7%	8–7.9%	
Mesorectal excision %			
TME	60–69.7%	69–68.3%	1
PME	26–30.3%	32–31.7%	
Defunctioning Stoma %	32–37.3%	38–37.6%	1

### Outcomes

**Fig. 2:**
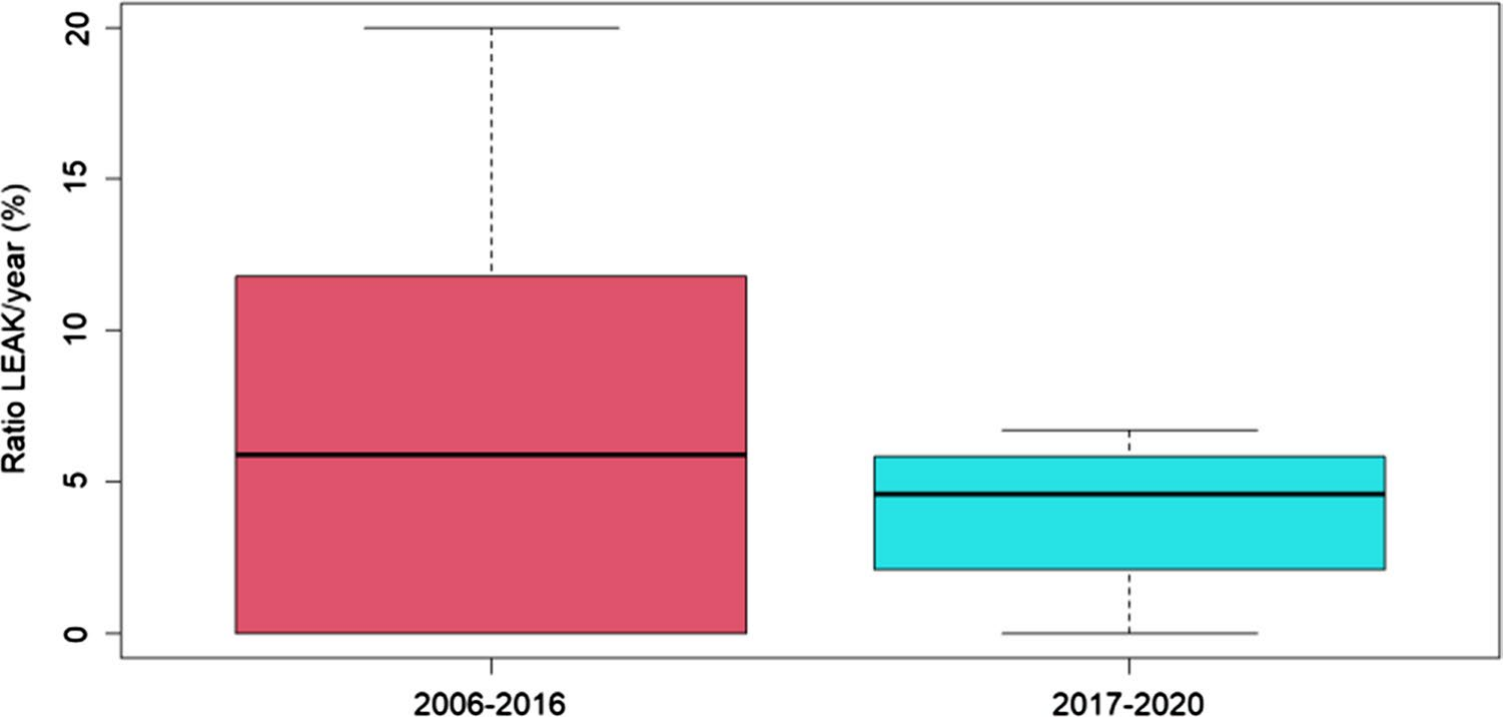
Ratio leak/year between Groups

Anastomotic leak was respectively 3.5% in Group A and 8.9% in Group B (p 0.149), higher but non-significant (Fig. 2).

However, estimated leak rate (2017–2026) for group A was significantly reduced when compared to group B (p 0.047) (Table 4).

**Table 4:** Anastomotic leak prediction

Leak Prediction	LEAK	no LEAK	P (0.047)
2006–2016	9	92	
2017–2026	7	221	

Operative time and the use of postoperative blood transfusion were significantly lower in Group A compared to group B: 190.3 ± 63 vs 220.7 ± 70.8 (p 0.002) and 11.6 vs 24.7% (p 0.002). Also, there was a significant higher use of laparoscopy in group A vs group B: 52.3 vs 19.8% (p 0.0002) and a reduction in the conversion to open surgery: 8.1 vs 28.5% (p 0.002). Mean and median LOS for group A and B were found to be significantly different: 6.5 ± 3.8 vs 12 ± 10.5 and 5 vs 9 days respectively (p 0.001).

No differences were found between Groups in overall postoperative complications, SSI, pneumonia, bleeding, readmission rate, mortality, 1-year stoma persistence and reoperation rate.

Three anastomotic leaks were detected in Group A: one late leak grade B in a patient who already had a protective loop ileostomy fashioned at index operation, was treated conservatively; another early leak grade A was also treated conservatively, whilst the third was a C grade leak requiring reoperation, abdominal lavage and a defunctioning loop colostomy. Both patients have loop ileostomy and colostomy closure after 9 and 33 months respectively.

In Group B, nine leaks were observed (one grade A, one grade B, seven grade C): two patients with diverting stomas developed leaks grade A and B and were treated conservatively; three patients with early leaks grade C who also had a loop ileostomy, required reoperation, abdominal lavage and drainage. Four patients with grade C leaks also required surgery: in two cases the anastomosis was saved and lateral colostomy fashioned; in two cases the rectal stump sutured and an end-colostomy created. Out of the eight patients having a defunctioning stoma, four were never recanalized while the other four after 2, 7, 9 and 12 months. No leak-related mortality was observed in both Groups.

One patient died (massive bowel ischemia) in Group A and two in Group B (one patient had an haemorrhagic shock and one suffered of heart failure).

Outcomes results are summarized in Table 5.

**Table 5:** Results of primary and secondary outcomes

Parameters	Group A (rectal cancer 2017–2020) (n = 86)	Group B (rectal cancer 2006–2016) (n = 101)	P
Operative Time (min) (mean, SD)	190.3 ± 63	220.7 ± 70.8	0.002
Need for conversion to open %	4/49–8.1%	8/28–28.5%	0.002
Postoperative blood transfusion %	10–11.6%	25–24.7%	0.024
Radicality %			0.552
R1	4–4.7%	7–6.9%	
R2	0–0%	0–0%	
Postoperative length hospital stay (days)			0.001
(mean, SD) (median)	6.5 ± 3.8 5	12 ± 10.5 9	
Complications % n patient	17–19.7%	29–28.7%	0.176
Anastomotic leak	3–3.48%	9–8.9%	0.149
Ileus	10–8.6%	19–18.8%	0.228
SSI	6–7%	10–9.9%	0.603
Pneumonia	4–4.6%	7–6.9%	0.552
Bleeding	1–1.2%	5–5%	0.220
Clavien-Dindo %			0.555
0	63–73.3%	62–61.4%	
1	4–4.7%	10–9.9%	
2	12–13.9%	17–16.8%	
3a	3–3.5%	3–3%	
3b	3–3.5%	5–5%	
4	0–0%	2–2%	
5	1–1.2%	2–2%	
Reoperation rate %	3–3.5%	10–9.9%	0.147
30 days readmission rate %	2–2.3%	3–3%	1
30 days mortality %	1–1.2%	2–2%	1
1-year stoma persistence %	9/33–27.2%	18/43–41.8%	0.231

## Discussion

This study analyzes how anastomotic leak and perioperative outcomes of patients undergoing anterior rectal resection for cancer are influenced by a significant increase in surgical volume at the same institution.

The main result of this study is the demonstration of a significant reduction in estimated anastomotic leak rate following ARR possibly due to centralization of service within the same Hospital.

A correlation between volume and anastomotic leak rates has been reported in only two other studies in the literature: a metanalysis by Huo et al., including 15,446 patients, demonstrated a significant reduction in anastomotic leak rate for rectal surgery with a HR of 0.75 (CI 95% 0.58–0.97) while, on the contrary, in a Dutch series, anastomotic leak was decreased in low-volume Hospitals for T1-3 rectal cancers [37, 38]. Other two notable studies are the large series by El Amrani et al. and Burns et al. [39, 40]. The former analyzed 45.569 rectal cancer resections performed in France between 2012 and 2016 and could not demonstrate a correlation between volume and anastomotic dehiscence. The latter identified 109.261 patients undergoing colorectal resection and again no significant association between surgical volume and leak was found. Overall the existing literature is inconclusive on this aspect.

Our study brings new data in favor of a positive volume-outcome correlation in rectal cancer surgery in a single institution. Between January 2006 and November 2016 five surgeons in three surgical units within the same large, tertiary academic center were operating on rectal cancer patients. In November 2016, a decision to centralize rectal cancer patients to only one surgical unit was taken. The decision was taken after an internal audit, in accordance between surgical units and with full support from the top. The audit served as a baseline assessment and guided further developments. A core team was established, with common perspectives and objectives. Interactions with other specialist units, including Gastroenterology, Oncology, Radiology and Radiotherapy were strengthened through more frequent, specifically dedicated multi-disciplinary meetings. Furthermore, in order to increase colorectal cancer case referral to the unit, a closer collaboration with General Practitioners (GP) was started by seeking out local GPs to inform them and making them active contributors to the project. Operational preparation and strategic planning helped building strong referral networks and, at the same time, the use of laparoscopy and implementation of enhanced recovery pathways was promoted.

Patients in the two groups were comparable for what concern known risk factors for anastomotic dehiscence, such us diabetes, smoking attitude, etc. [41, 42]. A few confounders may be pointed out but most of them are not expected to influence results significantly. The two cohorts are separated in time and it could be argued that change of practice other than volume might have resulted in the observed results. Yet, leak rates after ARR have been reported unchanged throughout the literature for many years and no reproducible significant improvement was introduced in clinical practice between 2006 and 2020, with the possible exception of indocyanine green, which in any case was not used in this series [43, 44]. The two groups, despite being relatively homogeneous, differ in some respects. A fast-track protocol has been implemented in the second time period (resulting in fact in lower LOS) but it has been widely demonstrated that ERAS has no impact on anastomotic leak [19, 20]. The same can be asserted for laparoscopy, which can reduce other postoperative complications such us surgical site infections, but does not affect dehiscence rate [14–16].

Depth of invasion (T stage) was greater in Group A, suggesting a surgery generally more demanding and more prone to complications in Group A, therefore adding value to the results. Finally, patients in Group B tended to have higher preoperative hemoglobin values and a higher need for transfusions, which may be interpreted as an increased intra- post-operative bleeding. This, indeed, may have favored Group A, as acute anemia is a known risk factor for anastomotic fistula [45, 46]. Yet, this may be justified with improved intra and postoperative surgical and anesthesiological management, referable to increased volume.

As Group A spanned a much smaller number of years than Group B, prediction of estimated leak rate for the years 2021–2026 was needed to render the two groups susceptible to (statistically) more appropriate comparison.

Anastomotic leakage is a very severe complication after ARR, often treated multidisciplinary with a reported rate up to 13.4% in an early phase and around 20% in the long-term [47]. Generally, mortality rate is low and, in our series, we did not report any leak-related mortality even though it has been reported to be up to 20% in some studies [21, 22, 27, 47]. Besides mortality, anastomotic leak has a significant impact on long-term functional outcome with the risks of sphincterial function loss and an unintended permanent stoma rate around 20% and it has a significant impact in quality of life and costs for health system [23–27].

Rationale in favor of case centralization to high volume centers is based on two consideration. First, rectal cancer management is particularly complex in many respects, needing multidisciplinary evaluation, availability of numerous treatment options, specialist nurses, teamwork and possibly research facilities [48–50]. Furthermore, it is often a complex procedure requiring highly skilled surgeons to perform meticulous dissection in the narrow space of the pelvis, using minimally invasive techniques.

On the other hand, the evidence regarding volume/outcome is still too conflicting to draw a conclusion in favor of centralization [51].

Nevertheless, some evidences in favor of a better outcome in high volume facilities comes from multiple large series and metanalysis. The most relevant finding is a decreased 30-days mortality, which is reported in many series [36, 52, 53]. Overall morbidity is reduced in the series reported by El Amrani et al. [39]; Baek et al. report higher rate of sphincter saving procedures, and Jonker et al. found a higher rate of complete radical resection for T4 cancers [38, 53]. Aquina et al. argues that high volume surgeons in high volume hospitals only obtain the best results [52]. Examples of centralizations in Europe for rectal cancer surgery exists in the Netherlands, Germany, Ireland, Norway, Sweden and Spain [54–58].

Yet, in a Dutch series by Jonker et al., high volume hospitals perform worse than low volume in terms of complications and other equally large series also failed to find any significant differences between low and high-volume centers [11, 38, 59–61].

A big limitation in confronting these studies is the definition of “high volume” as there is no consensus on the cutoff value. Recent UK guidelines specifically on this topic could not define a threshold because evidence is not strong enough to set one, since it would mean to cut out hospitals currently performing fewer procedure without a certain justification [12].

Another aspect is whether the surgeon rather than the center should be the target of centralization. Although some investigators reported better outcome in high volume surgeons, studies so far have shown a high variation in outcomes with mortality ranging from 0 to 7.7% suggesting that high volume per se*,* not supported by adequate structures and investments, could not be sufficient to improve outcome [61]. As a matter of fact, current guidelines recommend treatment in centers that can provide standard of care management including multidisciplinary approach (with dedicated oncologists, radiologists, radiation oncologists, endoscopists, surgeons and specialist nurses) but do not make any recommendation regarding hospital volume [13].

Other finding which would testimony on the benefits of volume increase are reduction in LOS, mean operative time, blood transfusion and need of conversion to open surgery. However, giving the retrospective nature of the study and the different time spam in which the two groups of patients were operated, it cannot be clarified whether these results are in effect due to other factors such as the implementation of ERAS and laparoscopy during the years. It is possible that the increase in volume reduces operative time, need for conversion to open surgery and postoperative blood transfusions; LOS could be correlated to both ERAS and minimally invasive surgery approach [15, 20, 62–66]. Other secondary outcomes such as postoperative complications, readmission rate, reoperation and mortality rate and 1-year stoma persistence were not significant in this study. It is unknown whether this could be due to the low accrual.

Overall, this study seems to add new information on the effect of volume on rectal surgery.

To the best of our knowledge, this is the first study evaluating effects of surgical volume in rectal cancer perioperative outcomes within the same Hospital. Therefore, it is pioneering in investigating effects of increasing volume in one department, rather than comparing different centers with different contexts, in an effort to isolate volume effect and minimize bias due to sample heterogeneity. Most studies in the literature come from population registry studies, involving many different hospitals and many different surgeons within the same Hospital, assembling a heterogeneous jumble of different practices in pre-, intra- and post-operative care. Patients in this study represent a homogenous group of patients managed in the same institution with uniform practices regarding protocols of treatment (e.g. neoadjuvant therapy), and postoperative management (e.g. early detection of leak with serial blood tests, CT scan with contrast per rectum, conservative management if possible). This potentially eliminates bias of comparing different situations, as is the case with population registries.

Furthermore, although being a study on surgical volume, rather than supporting “centralization” of care to pre-existing high volume units, results from this study embrace the possibility of “potentiation” of surgical units already present to optimize results, in line with the current literature.

Some limitations to this study are acknowledged by the authors. In particular, the retrospective nature of the study limit reliability of results, the number of patients treated is relatively low representing a potential bias and the single center experience is not easily generalizable to other centers. Oncologic outcomes have not been analyzed in the present study for lack of sufficient follow up for the most recent cohort of patients.

## Conclusion

Our study demonstrates that in response to an increased volume of ARR procedures for rectal cancer in a surgical unit, postoperative estimated anastomotic leak rate was significantly reduced despite treatment of more complex cases. Potentiation of existing low/medium volume surgical departments able to provide standard care for rectal cancer, by operational preparation and strategic planning, can yield improved perioperative results.

## Data Availability

Data supporting reported results can be found in the database of Policlinico Tor Vergata (www.ptvonline.it). Data are protected and access availability must be obtained.
